# Health impacts of PM_2.5_ originating from residential wood combustion in four nordic cities

**DOI:** 10.1186/s12889-022-13622-x

**Published:** 2022-07-04

**Authors:** Hans Orru, Henrik Olstrup, Jaakko Kukkonen, Susana López-Aparicio, David Segersson, Camilla Geels, Tanel Tamm, Kari Riikonen, Androniki Maragkidou, Torben Sigsgaard, Jørgen Brandt, Henrik Grythe, Bertil Forsberg

**Affiliations:** 1 Umeå University, Sustainable Health, 901 87 Umeå, Sweden; 2 University of Tartu, Ravila 19, 50411 Tartu, Estonia; 3 Finnish Meteorological Institute, P.O. Box 503, Erik Palménin aukio 1, 00101 Helsinki, Finland; 4 Centre for Atmospheric and Climate Physics Research, and Centre for Climate Change Research, University of Hertfordshire; College Lane, AL10 9AB Hatfield, UK; 5 Norwegian Institute for Air Research, Instituttveien 18, P.O. Box 100, 2027 Kjeller, Norway; 6 Swedish Meteorological and Hydrological Institute, SE-60176 Norrköping, Sweden; 7 Department of Environmental Science, Aarhus University, Frederiksborgvej 399, 4000 Roskilde, Denmark; 8 Department of Public Health , Aarhus University, Bartholins Allé 2, 8000 Aarhus, Denmark; 9 iClimate – interdisciplinary Centre for Climate Change, Aarhus University, Frederiksborgvej 399, 4000 Roskilde, Denmark

**Keywords:** Air pollution, Wood smoke, Premature death, Northern Europe, Life expectancy

## Abstract

**Background:**

Residential wood combustion (RWC) is one of the largest sources of fine particles (PM_2.5_) in the Nordic cities. The current study aims to calculate the related health effects in four studied city areas in Sweden, Finland, Norway, and Denmark.

**Methods:**

Health impact assessment (HIA) was employed as the methodology to quantify the health burden. Firstly, the RWC induced annual average PM_2.5_ concentrations from local sources were estimated with air pollution dispersion modelling. Secondly, the baseline mortality rates were retrieved from the national health registers. Thirdly, the concentration-response function from a previous epidemiological study was applied. For the health impact calculations, the WHO-developed tool AirQ + was used.

**Results:**

Amongst the studied city areas, the local RWC induced PM_2.5_ concentration was lowest in the Helsinki Metropolitan Area (population-weighted annual average concentration 0.46 µg m^− 3^) and highest in Oslo (2.77 µg m^− 3^). Each year, particulate matter attributed to RWC caused around 19 premature deaths in Umeå (95% CI: 8–29), 85 in the Helsinki Metropolitan Area (95% CI: 35–129), 78 in Copenhagen (95% CI: 33–118), and 232 premature deaths in Oslo (95% CI: 97–346). The average loss of life years per premature death case was approximately ten years; however, in the whole population, this reflects on average a decrease in life expectancy by 0.25 (0.10–0.36) years. In terms of the relative contributions in cities, life expectancy will be decreased by 0.10 (95% CI: 0.05–0.16), 0.18 (95% CI: 0.07–0.28), 0.22 (95% CI: 0.09–0.33) and 0.63 (95% CI: 0.26–0.96) years in the Helsinki Metropolitan Area, Umeå, Copenhagen and Oslo respectively. The number of years of life lost was lowest in Umeå (172, 95% CI: 71–260) and highest in Oslo (2458, 95% CI: 1033–3669).

**Conclusions:**

All four Nordic city areas have a substantial amount of domestic heating, and RWC is one of the most significant sources of PM_2.5_. This implicates a substantial predicted impact on public health in terms of premature mortality. Thus, several public health measures are needed to reduce the RWC emissions.

## Background

The use of biomass combustion for heating and energy production was the first ever fuel used by mankind, and it is still being widely used [1]. Currently, biomass constitutes approximately 12% of the global energy supply [2]. Biomass burning is a significant source of air pollution that has global, regional, and local impacts on air quality, public health, and climate (e.g. black carbon) [3]. It is well established that exposure to air pollution, in general, constitutes a serious global health risk [4]. Air pollution exposure constitutes a serious global health risk, and it was the fourth leading risk factor for deaths and disability-adjusted life-years in 2019 [5, 6]. There has been a large number of studies focusing on both short- and long-term health effects associated with air pollution exposure, and with a special focus on exposure to particulate matter (PM_10_) and/or fine particles (PM_2.5_) [7, 8].

Households have traditionally been using biomass of wood logs, crop residues, weeds, branches, and leaves for cooking and heating, and this procedure is still being used in a number of rural areas [9]. Residential wood combustion (RWC) is also common in many areas in the developed world [10]. This is especially true in those regions where a large supply of firewood is available for household heating during wintertime [11]. Recently, the European Union has exerted high pressure on several member states to ensure that the wood usage will fulfil the renewable energy obligations under the Paris Agreement [12].

During wood combustion, a number of harmful pollutants including carbon monoxide (CO), volatile organic hydrocarbons (VOC), polycyclic aromatic hydrocarbons (PAH), PM_10_, and PM_2.5_ are released [13]. These emitted particles can be divided into three different categories; inorganic ash materials, soot (black carbon), and condensed organic materials. The emissions of these pollutants are mainly caused by incomplete combustion where the emissions are highly dependent on the combustion efficiency [14]. The mass distribution of particles originated from wood peaks at a particle diameter of approximately 0.1 − 0.2 μm [15], and they are classified as ultrafine particles (size < 100 nm) [16].

Small-scale wood combustion (i.e., combustion caused by stationary small-scale appliances used in homes, and small- and medium-scale industries, etc.) that occurs mainly in Europe, Asia, and Africa is responsible for emitting a large amount of atmospheric particulate matter [3, 17]. Globally, the contribution of domestic fuel burning to the mass of PM_2.5_ is approximately 20% [18]. For Europe, the corresponding percentage contributions are 32%, 22%, 15%, and 12% for Central and Eastern Europe, North-western Europe, Western Europe, and South-western Europe, respectively [18].

The toxicity of RWC has been shown in both epidemiological and toxicological studies. The epidemiological studies have mainly focused on cardio-pulmonary morbidity and mortality [19], but other outcomes (e.g., dementia) have also been studied [20]. Many of the toxicological studies are chamber studies where healthy individuals have been exposed to wood smoke and clean air. In one of these studies, blood samples and urine samples were taken before and after their exposure sessions [21]. The markers that were analyzed indicated that exposure to wood smoke affects inflammation, coagulation, and possibly lipid peroxidation. In the recent STOVES study, young healthy participants were exposed to wood smoke from different cook stoves for two hours. Acute effects on inflammatory biomarkers, blood lipids, and heart rate variability were reported even though some of the measured outcomes showed inconsistencies with no clear exposure-response associations e.g. [22–24]. However, based on a recent review that included 22 identified publications, consistency of the results regarding the effects on the airways was recognized, whereas for oxidative stress, systemic inflammation, and cardiovascular physiology, no clear patterns were found [25]. In a position paper that discussed the contribution of biomass combustion to pollution concentrations in Europe, and the emerging evidence of adverse health effects, wood smoke was estimated to cause at least 40 000 premature deaths per year in Europe [26]. Many studies indicate that the combustion-related particles are especially important in terms of harmful health effects [27, 28], and that they might pose a higher risk than other particles [29]. Furthermore, as a result of coordinated interventions that reduced wood smoke emissions in Tasmania in Australia, decreases both in terms of all cause, cardiovascular, and respiratory mortality occurred [30].

In a previous study by Kukkonen et al. [31], the emissions originating from RWC were thoroughly analyzed together with their atmospheric dispersion in four target city areas (i.e., Umeå, Helsinki, Copenhagen, and Oslo). The modelled concentrations were also evaluated against air quality measurements. Our current study extends the analyzes in Kukkonen et al. [31] by calculating the health effects associated with exposure to local emissions of RWC induced PM_2.5_ in these four Nordic city areas. These city areas have a high amount of domestic heating, and RWC is one of the largest local sources of fine particles. With our current analysis, we aim to estimate the health effects of the population based on (i) RWC induced air pollution exposure, (ii) recent mortality data taken from the national health registers, and (iii) concentration-response functions (CRF) derived from a previous epidemiological study. More specifically, we intend to calculate (i) the number of premature deaths, (ii) years of life lost (YLL), (iii) average YLL per premature death case, and (iv) decrease in life expectancy.

## Methods

### Study areas

This study focuses on three Nordic capital regions: Helsinki Metropolitan Area, Oslo, and Copenhagen, and in Sweden on the smaller city of Umeå (where detailed exposure data were currently available), and their neighboring areas. We will focus on the larger urban areas instead of solely focusing on the areas of the cities themselves. For instance, we examine the four separate cities, which together comprise the Helsinki Metropolitan Area, as well as Umeå and its neighboring villages. However, we will subsequently refer to the regions simply as Helsinki, Oslo, Copenhagen, and Umeå. The locations of the selected cities and their domains are thoroughly presented in an earlier analysis by Kukkonen et al. [31].

### Air pollution exposure assessment

The population exposure to RWC induced air pollution from local sources is based on both the modelled annual average PM_2.5_ concentration and the population density in different parts of the urban areas. PM_2.5_ has been chosen as a comprehensive indicator of several pollutants originating from RWC [31, 32]. For the air pollution modelling, the following information was collected from three sources: (i) wood usage for combustion and combustion appliances sources (details are presented in the earlier analysis by Kukkonen et al. [31]), (ii) emissions factors, and (iii) temporal allocation of emissions. The assessment of emissions from RWC for these four cities is also described by Kukkonen et al. [31]. The RWC induced PM_2.5_ concentrations were calculated by using air pollution modelling.

The spatial resolution (size of grid-square cells) was 250 m × 250 m for Umeå and Helsinki, and 1 km × 1 km for Oslo and Copenhagen. The results represent the year 2011 for Umeå, 2013 for Helsinki and Oslo, and 2014 for Copenhagen. All four cities used different dispersion models: DISPERSION for Umeå (a multiple-source Gaussian model), UDM-FMI and SILAM for Helsinki (a multiple-source Gaussian model, and a global and regional scale chemical transport model), EPISODE for Oslo (a 3D Eulerian and Lagrangian model), and DEHM/UBM for Copenhagen (a 3D Eulerian and Gaussian models). Details of the models are presented in the earlier analysis by Kukkonen et al. [31].

For Helsinki, the gridded population data were obtained from the population grid of the Helsinki Metropolitan Area [33]. The population grid consists of grid-based statistics of this region that include information of the total population, the population density, and the age distribution of the population. In this study, the population data of 2018 were used. For Oslo, the 2013 gridded population data from Statistics Norway were used. The dataset was based on information from the national building and population registries. In addition, this dataset was combined with 2013 population data by age and gender in Oslo from Statistics Norway. For Copenhagen, the population data were obtained from the central personal register (CPR) for the year 2014, and aggregated to the 1 km x 1 km resolution. For Umeå, gridded population data for different age groups with a resolution of 100 m x 100 m were provided by Statistics Sweden for the year 2011.

**Fig. 1:**
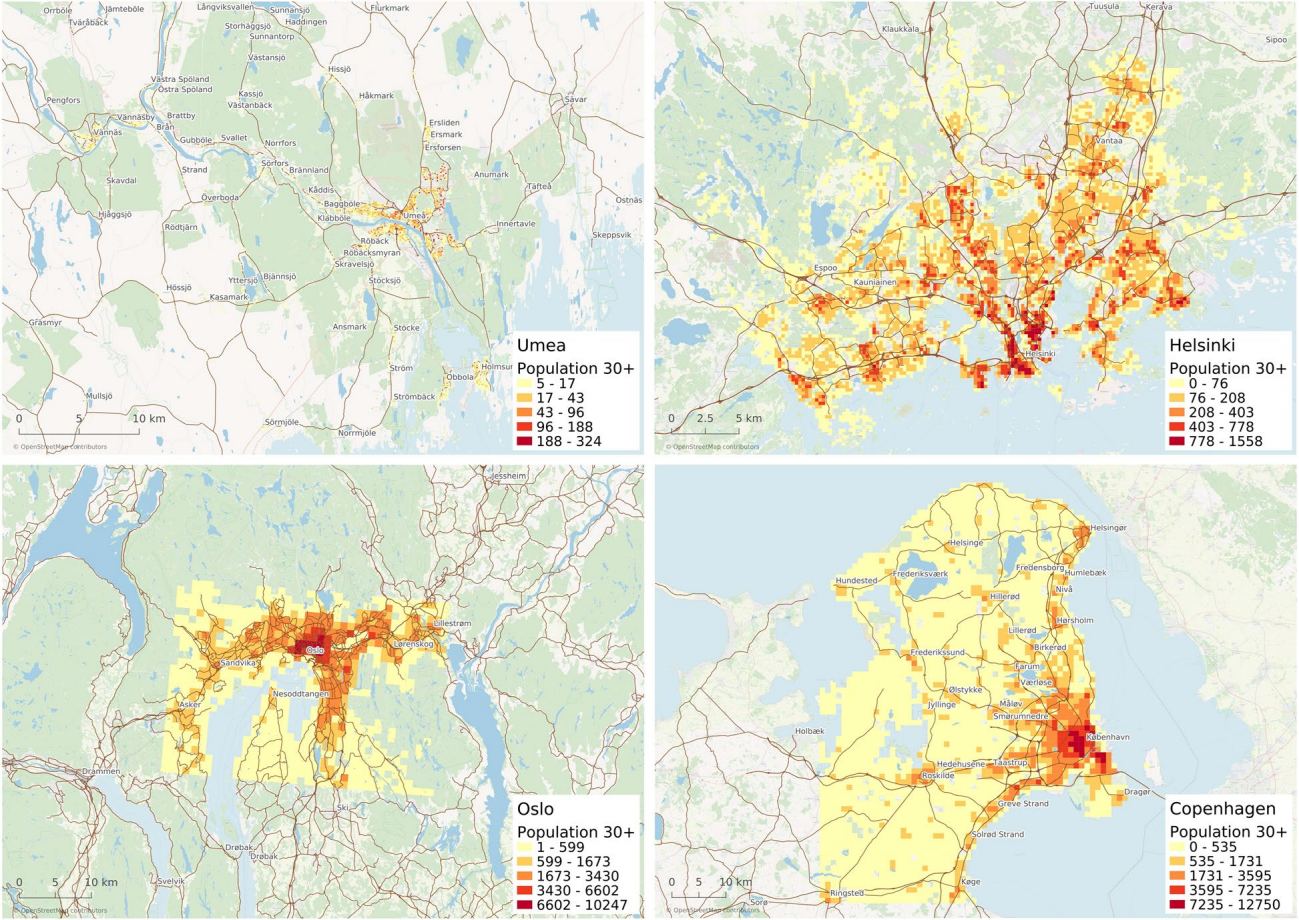
The population density for the population aged 30 years and older. The spatial resolution is 250 m × 250 m for Umeå and Helsinki, and 1 km × 1 km for Oslo and Copenhagen. The results represent the year 2011 for Umeå, 2018 for Helsinki, 2013 for Oslo, and 2014 for Copenhagen. Maps created with QGIS 3.14.1-Pi, https://qgis.org/en/site

Based on these data (Fig. 1), the population-weighted concentration (C_pop_) of RWC in each city was calculated. For this, the calculated concentration in each grid-square cell (Ci) was multiplied with the number of people in the corresponding grid-square cell (Pi), and then all products were summed. The result of this sum was finally divided by the total population (Eq. 1).

1 $$Cpop=\frac{\sum Ci Pi}{\sum Pi}$$

Using this principle, the grids with a higher population density give a larger contribution to the population-weighted mean concentration.

### Calculation of health impacts

The numbers of premature deaths were calculated based on health impact assessment (HIA) principles. HIA combines the results of RWC air pollution exposure, baseline mortality in the population, and concentration-response functions (CRF) from earlier epidemiological studies. The baseline mortality data in the population were retrieved from Statistics Sweden [34], Statistics Finland [35], Statistics Norway [36], and Statistics Denmark [37]. We applied the most recent total mortality data (2019) in the age group 30+. Considering that the health impact calculations apply to Nordic cities, we employed CRF from Hvidtfeldt et al.‘s [38] study from Denmark. This resulted in a Hazard Ratio (HR) of 1.26 (95% CI: 1.10–1.42) per 10 µg m^− 3^ increment in PM_2.5_ for all-cause mortality. Moreover, this HR is in line with Turner et al. [39] who found a HR of 1.26 (95% CI: 1.19–1.34) per 10 µg m^− 3^ increase in near-source PM_2.5_ in a large study using cohort data from the American Cancer Society Cancer Prevention Study II (ACS CPS II).

AirQ + was used for the calculation of the following four variables: (i) the number of premature deaths, (ii) the years of life lost (YLL), (iii) the average YLL per premature death case, and (iv) the decrease in life expectancy. AirQ+, developed by the WHO, is a software tool for determining health risk assessment of air pollution exposure [40]. It has previously been used for the Nordic area, and it has also been compared and validated against other similar tools [41]. It enables users to apply (i) concentration-response functions for selected air pollutants, (ii) data regarding air quality, and (iii) data regarding the population in terms of population size, age distribution, and baseline mortality. With these data entered into AirQ+, it is possible to calculate (i) the number of premature deaths, (ii) YLL, and (iii) decrease in life expectancy (i.e. the health effects from exposure to air pollutants) using different cut-offs and CRFs.

In this study, the health effects were calculated for the population aged 30 years and older, as no effects on premature mortality have been noticed among younger ages [42]. No cut-off concentrations were used as combustion particles are expected to be equally toxic at low concentrations. This principle has been applied in recent similar studies [43].

## Results

**Fig. 2:**
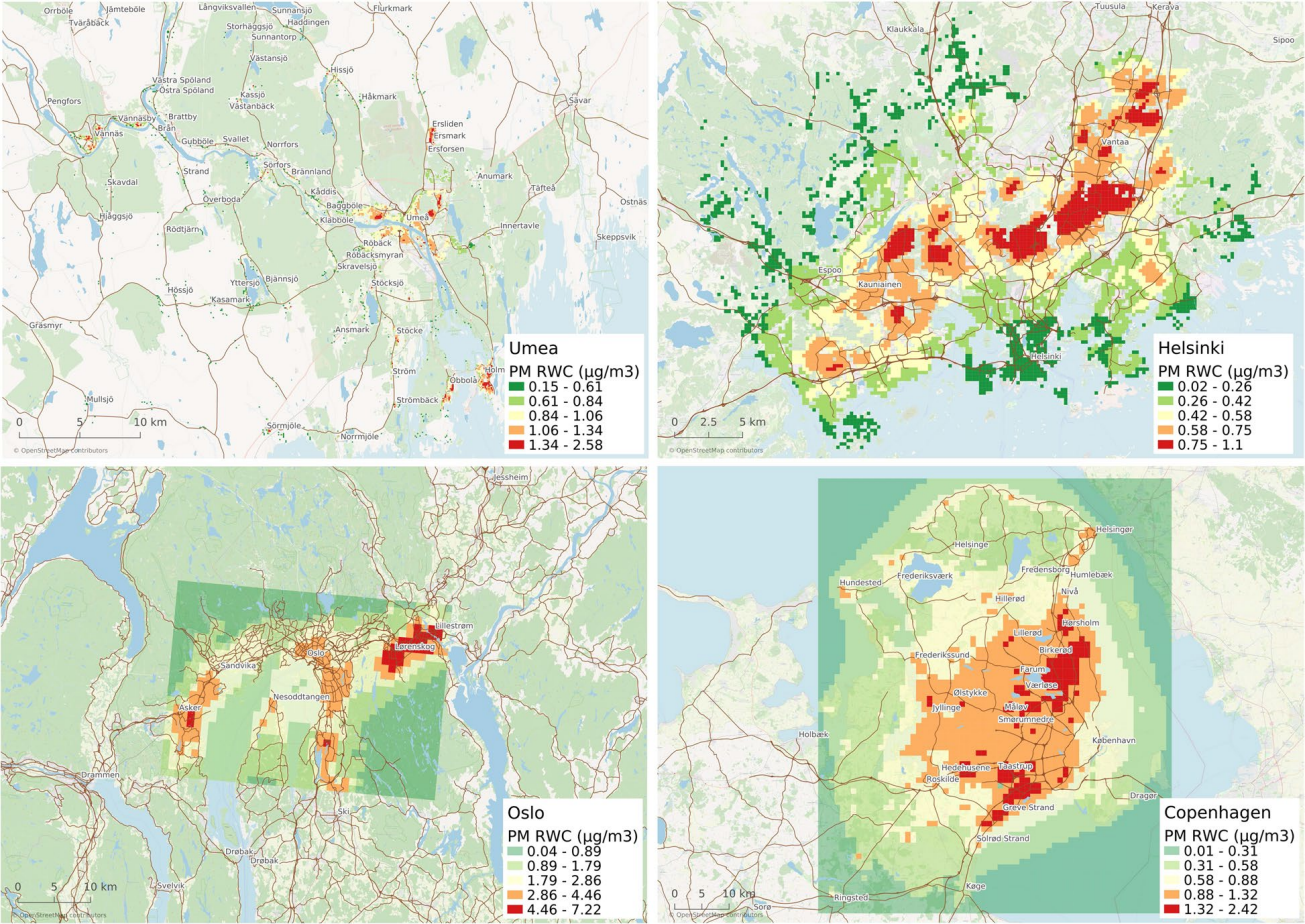
Annual average concentration of PM_2.5_ due to emissions from RWC in Umeå, Helsinki, Oslo, and Copenhagen. The spatial resolution is 250 m × 250 m for Umeå and Helsinki, and 1 km × 1 km for Oslo and Copenhagen. The results represent Umeå (2011), Helsinki and Oslo (2013), and Copenhagen (2014). The physical sizes of the domains are different for each panel. Maps created with QGIS 3.14.1-Pi, https://qgis.org/en/site

Among the four studied cities, the contribution of RWC induced PM_2.5_ from local sources was highest in the Oslo metropolitan where the maximum annual average concentration of PM_2.5_ due to emissions from RWC is 7.22 µg m^− 3^ (Fig. 2). Among the four studied cities, the highest annual average PM_2.5_ concentration due to emissions from RWC was detected in Oslo (6.68 µg m^− 3^), followed by Umea (2.58 µg m^− 3^), Copenhagen (2.42 µg m^− 3^) and Helsinki (1.10 µg m^− 3^) (Fig. 2). However, there were high spatial variations as the annual average PM_2.5_ concentration due to emissions from RWC in some areas of Copenhagen was as low as 0.01 µg m^− 3^. Taking the population distribution into account, the population-weighted annual average concentrations of RWC induced PM_2.5_ were 0.93 µg m^− 3^ in Umeå, 0.46 µg m^− 3^ in Helsinki, 2.77 µg m^− 3^ in Oslo, and 0.98 µg m^− 3^ in Copenhagen (Table 1).

**Fig. 3:**
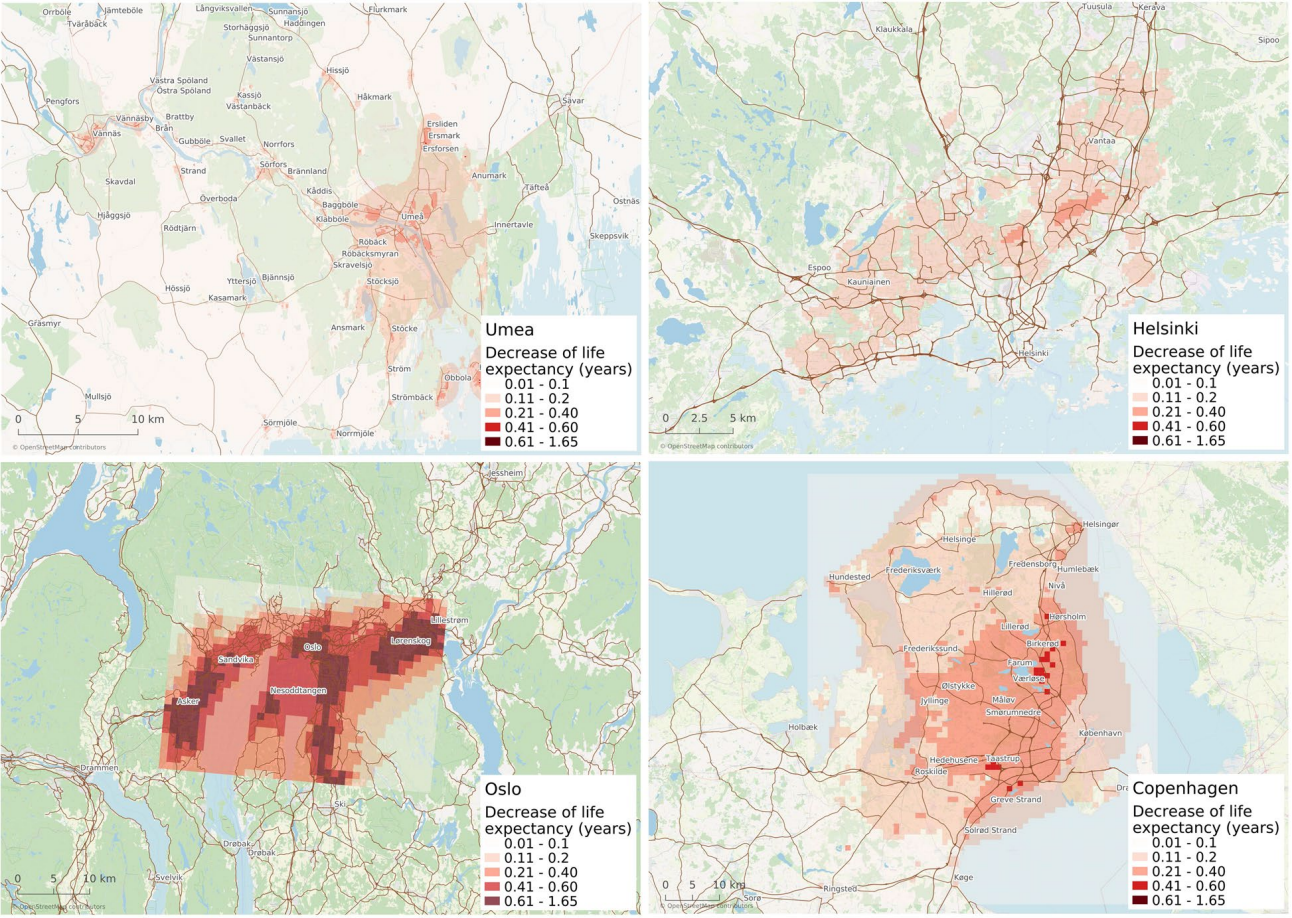
The decrease in life expectancy in different parts of the city areas attributed to the concentrations of PM_2.5_ originating from RWC. Maps created with QGIS 3.14.1-Pi, https://qgis.org/en/site

Due to having the highest annual average RWC induced PM_2.5_ concentrations, the health effects associated with RWC were largest in Oslo with an average decrease in life expectancy by 0.63 (95% CI: 0.26–0.96) years (Table 1). The effects were lowest in Helsinki where the average decrease in life expectancy was 0.10 (95% CI: 0.05–0.16) years. The corresponding numbers in Umeå and Copenhagen were 0.18 (95% CI: 0.07–0.28) and 0.22 (95% CI: 0.09–0.33) years respectively. The health effects were larger in certain areas of the cities with a higher proportion of RWC. Subsequently, this effect was especially prominent in Oslo and Copenhagen (Fig. 3).

**Table 1:** Data regarding the study areas (column 1), population-weighted annual average concentrations of local emissions of PM_2.5_ from RWC in the age group 30+ (column 2), the number of inhabitants in the age group 30 + in each study area (column 3), and the health impacts calculated in AirQ+ (columns 4 − 7)

Study area and the year examined^a^	Population-weighted annual average concentration in the age group 30+ (µg m^− 3^)	Number of inhabitants in the age group 30+	Premature death cases in one year due to RWC exposure (95% CI)	Decrease in life expectancy (95% CI), years	Average loss per premature death case, years	Years of Life Lost (95% CI) in one year
**Umeå (2011)**	0.93	76,204	19 (8–29)	0.18 (0.07–0.28)	9.0	172 (71–260)
**Helsinki (2013)**	0.46	759,127	85 (35–129)	0.10 (0.05–0.16)	10.1	824 (351–1216)
**Oslo (2013)**	2.77	416,316	232 (97–346)	0.63 (0.26–0.96)	10.6	2,458 (1,033–3,669)
**Copenhagen (2014)**	0.98	632,255	78 (33–118)	0.22 (0.09–0.33)	10.2	794 (330–1,198)
**The sum or population weighted average of all four cities**	1.16	1,883,902	414 (173–622)	0.25 (0.10–0.36)	10.2	4,248 (1,785–6,343)

^a^ The health impacts are based on population-weighted concentrations of PM_2.5_ from RWC for a certain one-year period in each study area

Due to the high exposure to PM_2.5_ from RWC and the relatively large number of inhabitants (416,316 persons in the age group 30 + years old), a total of 232 (95% CI: 97–346) premature deaths are expected in Oslo annually. The corresponding number of premature deaths caused by exposure to RWC is 78 (95% CI: 33–118) in Copenhagen, 85 (95% CI: 35–129) in Helsinki, and 19 (95% CI: 8–29) in Umeå. The number of deaths is lowest in Umeå due to a relatively small population. The average decrease in life expectancy per premature death in the four city areas is approximately ten years which is similar in all four city areas. The numbers of YLL follow the same trend as the numbers of premature deaths with a total sum of 4,248 years (Table 1)

## Discussion

### The calculated health impacts

The current study shows that RWC has a relatively high health burden in terms of premature deaths, years of life lost, and decrease in life expectancy. However, the health impacts vary in the four Nordic city areas. The average annual estimates of the number of premature deaths range from 19 (Umeå) up to 232 (Oslo), and these estimates are reflected by differences in (i) the amount of exposure to PM_2.5_ from RWC, (ii) the population size, and (iii) the age distribution (e.g. proportion of elderly). The average decrease in life expectancy also varies, but significantly less than the number of premature deaths. This is because life expectancy is largely determined by the amount of exposure to PM_2.5_ from RWC, and to some extent also by the age distribution. However, life expectancy is not affected by the total number of inhabitants.

In Finland, the health effects associated with exposure to PM_2.5_ from RWC in 2015 were calculated in an earlier study conducted by Savolahti et al. [44]. The concentrations of PM_2.5_ from RWC were in the range of 0.5 − 2 µg m^− 3^ in the proximity of most urban areas, resulting in approximately 31 premature deaths in the cities with > 200,000 inhabitants in 2015. This estimation is significantly lower than the results in this study with 53 premature deaths in Helsinki in 2013. The Helsinki region has approximately one million inhabitants which constitutes around three-quarters of such urban agglomerations in Finland that have more than 200,000 inhabitants. The main differences in these two assessments were the different emission and dispersion computations, and, most significantly, the fact that different RRs have been used. Savolahti et al. [44] applied a RR of 1.062 (95% CI: 1.040–1.083) per 10 µg m^− 3^ increase in PM_2.5_, whereas more recent HIAs, including our study (e.g. [43]), have applied significantly larger RRs for combustion particles. The trends in the concentrations of PM_2.5_ originating from residential wood combustion in Helsinki region have been analyzed by Kukkonen et al. [45] during a multidecadal period with a slightly increasing trend from the 1980’s to the mid-2010s, caused by the more widespread use of residential wood combustion in the area.

The choice of concentration-response function has a crucial importance for the health impacts calculations. In this current study, the health impact calculations are based on a HR of 1.26 (95% CI: 1.10–1.42) per 10 µg m^− 3^ increase in PM_2.5_ for all-cause mortality based on a 15-year exposure to PM_2.5_ at the residential addresses as according to Hvidtfeldt et al. [38]. Hvidtfeldt et al.‘s study collected detailed data on air pollution exposure, lifestyle factors, and socio-demography on a group of participants who lived in the areas of Copenhagen and Aarhus from 1997 to 2015. This hazard ratio is based on exposure to PM_2.5_ in general, and not exclusively on RWC induced PM_2.5_. As the aim of this study is to calculate the health effects exclusively caused by exposure to RWC induced PM_2.5_, the application of a general hazard ratio based on PM_2.5_ exposure is expected to create some uncertainty.

Recently, Vohra et al. [43] applied a low concentration RR of 1.129 (95% CI: 1.109–1.150) for all-cause mortality in all ages associated with a 10 µg m^− 3^ increase in PM_2.5_ exposure in order to calculate the global mortality from outdoor fine particle pollution generated by fossil fuel combustion. Another recent European multi-cohort study suggested a similar RR [46]. According to Vodonos et al. [29], a RR at low concentrations could be even higher (1.24, 95% CI: 1.08–1.40 per 10 µg m^− 3^ increase in a meta-regression restricted to studies with mean concentrations of PM_2.5_ below 10 µg m^− 3^). A higher meta-estimate for the RR at low concentrations is also reported by Chen and Hoek [47]. In addition, Turner et al. [39] found a similar increased risk (RR = 1.26, 95% CI: 1.19–1.34 per 10 µg m^− 3^ increase in PM_2.5_) in a large study using cohort data from the American Cancer Society Cancer Prevention Study II (ACS CPS II) which is comparable to the RR in Hvidtfeldt et al. [38] that we have applied in this current HIA.

In an earlier Swedish report from 2018, where the population’s exposure to air pollutants was calculated as annual average concentrations, the number of premature deaths in Sweden associated with exposure to RWC induced PM_2.5_ was estimated to be 935 (95% CI: 292 − 1577) in the age group 30 + during the year 2015 [48]. In this study, and based on earlier data from a subset of subjects from the American Cancer Society Cohort in Los Angeles County [49], a smaller concentration-response function (RR = 1.17, 95% CI: 1.05–1.30 per 10 µg m^− 3^ increase in PM_2.5_) was applied. Thus, our estimation is almost two times higher due to applying a higher RR.

Another question we address is whether wood-smoke particles pose different levels of risk compared to other ambient particles of similar size. In general, it is difficult to determine the difference between long-term exposure to RWC induced PM_2.5_ and long-term exposure to PM_2.5_ in original epidemiological studies. This is due to the fact that people are exposed to a mixture of fine particles from different sources. The difference in short-term health effects associated with exposure to RWC induced PM_10_ as well as to PM_10_ from point sources and mobile sources has been analyzed in Chile in the cities of Temuco and Pudahuel [50]. In Temuco, the main source of pollution was RWC, while in Pudahuel, the main sources of pollution were point sources and mobile sources. The findings of this study showed that the RRs for cardiovascular and respiratory mortality were slightly higher in Temuco as compared to Pudahuel.

In contrast, a study in the Estonian city of Tartu [51], where traffic and RWC induced particles were modelled separately, no association was found between RWC induced particles and health symptoms, although traffic-induced particles increased the odds of cardiac disease. As this study used self-reported health data, cross-sectional design, and modelled exposure data, conclusions should be taken with reservation and more epidemiological studies focusing specifically on RWC are needed. Furthermore, some experimental studies have found different effects. For example, Riddervold et al. [52] reported that wood smoke at a concentration normally found in a residential area can cause a mild inflammatory response. In contrast, Forchhammer et al. [53] did not find any effects either on markers of oxidative stress, DNA damage, cell adhesion, cytokines, or microvascular function in the same 20 atopic subjects.

Finally, in a review study with 22 identified publications based on the results from twelve studies on controlled human exposures to wood smoke, a range of different combustion conditions, exposure concentrations, and durations were applied. Different effects on the airways and the cardiovascular system as well as systemic endpoints were assessed. Large variations regarding study design in the analyzed studies make it difficult to draw any general conclusions. However, the findings were broadly consistent with respect to the effects on the airways, but there were no clear patterns regarding the effects on oxidative stress, systemic inflammation, and cardiovascular physiology [25].

### Exposure assessment

In the current analysis, we have used modelled RWC induced PM_2.5_ concentrations from local sources and applied annual average concentrations at home addresses. On the one hand, this approach is similar to the original epidemiological studies from which the concentration-response functions have been obtained. On the other hand, the real-life situations are much more complex, and there could be several uncertainties in those exposure estimations. Firstly, the models often apply relatively large grid-square cells. Secondly, these grid-square cells may vary in size in the different city areas. In Oslo and Copenhagen, a cruder modelling domain was used as compared to Helsinki and Umeå. If a more finely spaced receptor grid had been used for Oslo and Copenhagen, the predicted exposure and health effect values would have been somewhat higher. This effect is caused by both the positive spatial correlation of the emissions of RWC and the locations of the population in these cities.

The computations were done on a spatial resolution of 250 × 250 m for Umeå and Helsinki, and 1 × 1 km for Oslo and Copenhagen. We have selected the finest possible spatial resolutions for the computations for all four cities. However, the spatial resolution influences the predicted exposure and health values. Such impacts have been examined previously by Karvosenoja et al. [54] and Korhonen et al. [55]. Both studies showed that the predicted exposure values were lower for computations with a coarser spatial resolution. It is therefore essential to use a sufficiently fine model resolution in view of the assessment of health impacts. This is especially important for primary particles from emission sources at low emission heights. Moreover, for Copenhagen and Umea region a larger modelling domain is shown in order to describe the contribution from emissions outside the city in detail. Consequently, in the present study, we estimate that the use of different spatial resolutions (250 × 250 m or 1 × 1 km) is expected to result in a difference of less than 10% in the health impact estimates, based on the results by Korhonen et al. [54].

Another important aspect is to find the best possible proxy of human exposure. We have used air pollution concentrations at home addresses as a proxy. However, people are mobile and, thus, they are exposed during the day to air pollution concentrations at different locations (e.g. at home, at work, during shopping, whilst commuting, etc.). A higher spatial resolution of exposure data will not necessarily give a better estimation of the personal exposures, and using very high-resolution exposure data requires a spatio-temporal personal exposure model for estimating the exposure in different environments during the day, which is not available at the moment. Coarser resolution exposure data gives an average of the exposure during a day in the area at home and in the nearest surroundings.

The highest concentrations of RWC particles from local sources were found in Oslo. This concentration was, on average, three times larger than the corresponding values in Umeå and Copenhagen, and five times larger than in Helsinki. Clearly, all modelling results are dependent on the accuracy of the information of wood usage for combustion and the adopted emission coefficients for combustion appliances. The most common sources of information regarding firewood consumption are usage statistics and different questionnaires [56]. For instance, in Denmark, the wood usage has been estimated through questionnaires for approximately 6 000 households with wood stoves. In the current study, different years with different meteorology have been modelled where outdoor temperature and windiness might have affected the energy demand for heating, and, consequently, also the emissions and dispersions. However, Kukkonen et al. [31] have thoroughly described and evaluated the applied RWC emission inventories, and these contain the best available emission data in each of the target cities. The emission inventories and meteorological data in the current analysis partly correspond to different years in the target cities, selected based on the availability of relevant data. According to Kukkonen et al. [31], none of the considered years was rare in any of these cities in terms of the ambient temperatures. Although comparing the results from different years includes an uncertainty, the differences in relevant meteorological conditions were not substantial for the selected years. In addition, only small variations in the populations of the target cities occurred during the period from 2011 to 2014, and this is not considered to have any noticeable impact on the health impact estimates.

One limitation of this study is that we only focused on local emissions and local impacts. PM_2.5_ originating from wood burning is also long-range transported in the atmosphere where it can spread up to thousands of kilometres away from the emission source. Therefore, the emissions from these four cities also influence health outside the city areas, and wood burning outside the cities contributes to the health impacts within these cities. Another limitation of this study is that we addressed only ambient RWC concentrations, as we were not able to estimate the contribution of RWC to indoor air pollution. Previous studies have shown that in some regions, the particle concentrations can be more than two times higher in homes with residential stoves [57, 58]. According to Vicente et al. [59], this increase is especially high during open fireplace operation where PM_10_ concentrations can rise up to twelve times as compared to background concentrations. Moreover, candles are very often used during wintertime in Denmark [60], and a high concentration of candle induced PM_2.5_ has shown a mild inflammatory response among young asthmatics as a result of five hours of exposure [61]. Nevertheless, it has been discussed that addressing ambient and indoor RWC exposure as separate risk factors can lead to double counting due to their interrelated nature [44]. Therefore, in order to avoid double counting, the results of this study should not be combined with the burden of disease estimates of indoor RWC exposure. Infiltration of outdoor particles indoors can be significant even in well-insulated buildings due to the operation of windows and doors, and cracks in the building envelope and windows, and door frames [62]. Population exposure can therefore be significantly different, depending on the structure and ventilation of buildings. The infiltration factors of PM_2.5_ have been estimated to range from 0.47 to 0.59 in the Helsinki area [63].

### Factors affecting the RWC emissions

Controlled experiments with different types of wood stoves have resulted in significantly different emissions. Fuel moisture, charge size, feeding rate, and air ventilation are also important factors in terms of emissions of particles [64]; e.g. high moisture content fuels will result in increased PM_2.5_ emissions [62].

During a laboratory study, where different kinds of wood species were burned in a wood stove during different burning conditions, it was evidenced that the user of the stove could largely influence the emissions both by fuel selection and through the choice of burning conditions. There were three main factors that were crucial when it came to creating a complete combustion: (i) a proper amount of well-mixed oxygen present in relation to the fuel, (ii) a suitable temperature, and (iii) an optimal residence time of the fuel/oxygen mixture. There were, however, no significant differences regarding the emissions from different types of wood [65].

With respect to the amount of emissions, the age of the burning device was according to laboratory tests an important factor in which there were large variations in the emissions from different wood combustion appliances. In general, more modern devices with newer technology gave rise to lower PM emissions compared to older devices. However, there are large differences in the emissions that occur during laboratory conditions compared to the emissions that occur in real-life conditions. There are only a limited number of tests that have been conducted under real-life conditions. Further studies and a better understanding of the prevailing real-life conditions is, therefore, needed since the user’s behavior is an important factor regarding the amount of RWC emissions [66].

### Policy implications

In order to reduce the health effects caused by wood combustion, several policy measures should be applied. These could include stricter guidelines for air quality, emission reduction measures, and improvements of pre-processing, storage, and combustion practices to lessen the associated health impacts. However, there has been a historical misconception that wood smoke is something natural that does not cause any serious health effects [67]. Furthermore, even though wood has been considered earlier as a renewable fuel with climate benefits, the validity of this statement depends on forest management policies and several other factors [68].

Over the past years, biomass combustion for residential heating has been increasing, and globally, it has been projected to become the major source of primary particle emissions over the next 5 − 15 years [69]. It is also important to bear in mind that wood burning emits black carbon [70] which is a short-lived climate forcer (SLCF) with a warming effect [71]. Thus, urgent actions are needed.

One of the measures regulating air quality has been the Ambient Air Quality Directive (2008/50/EC), established by the European Union that entered into force in 2015. According to this Directive, the limit value for PM_2.5_ aims for a maximum concentration of 25 µg m^− 3^ as a yearly average in many parts of the EU. However, and directly based on scientific evidence on the health impacts of fine particulate matter, the health-based guideline issued by the WHO for the annually averaged PM_2.5_ concentration is 10 µg m^− 3^. These limit and guideline values are substantially higher than the concentrations originating from RWC that were found in this study (max 7.22 µg m^− 3^, but mostly < 1 µg m^− 3^). In addition, benzo(a)pyrene, which is emitted during small-scale wood burning [72], is restricted as a target value in the EU (the annual mean value may not exceed 1 ng m^− 3^). Those target values are exceeded in several countries in the EU [73].

Another type of regulation is emission control. The European legislation for solid fuel boilers (2015/1189/EU) and local space heaters (2015/1185/EU) include emission limit values that have to be met. Unfortunately, these requirements entered into force in 2020 for boilers and will enter into force in 2022 for local space heaters. Moreover, these regulations regulate new boilers, but not the existing ones. For the already existing boilers and wood stoves, filters can be installed into the chimneys by using different technologies (e.g. electrostatic (precipitator) filters, cyclones, etc.) [74].

Thirdly, several Nordic countries have implemented *scrapping payments* and *replacement subsidies* [75]. Those programs have provided grants to individual property owners for the replacement of boilers with new low-emissions Eco-labelled products. Some studies have also addressed the effectiveness of stove exchange programs in order to reduce the PM emissions and ambient concentrations with different outcomes depending on factors such as the level of implementation [76–79].

However, emissions from firewood stoves considerably depend on the user´s behavior and habits, and the quality of the firewood [65]. In fact, all four Nordic countries have been instructed/notified by information campaigns in terms of the use of wood stoves and the proper storage of fuels [31]. Therefore, information campaigns should also promote cleaner domestic burning practices [31] and certifications of the quality of firewood.

## Conclusions

The present study is one of the few multi-city investigations that systematically addresses the health impacts of RWC. All these four Nordic city areas have a substantial amount of domestic heating, and RWC is one of the largest emission categories of fine particles. In the exposure assessment, we could identify relatively large inter- and intra-city variations in the PM_2.5_ concentrations in the studied areas. The RWC induced PM_2.5_ concentrations were highest in Oslo, where the population-weighted annual average concentration was 2.77 µg m^− 3^, and the population-weighted annual average concentration was lowest in Helsinki (0.46 µg m^− 3^).

This resulted in substantial predicted impacts on public health that ranged from 19 (Umeå) to 232 (Oslo) premature deaths per annum. This effect was comparatively largest in Oslo (an average decrease in life expectancy of 0.63 years) and lowest in Helsinki (an average decrease in life expectancy of 0.10 years). Regarding the premature death cases, the average loss per premature death case was 10.2 years. Subsequently, this resulted in more than 4,000 years of life lost per annum in total in the four target cities. Thus, RWC has a major impact on public health.

The current legislation in Europe regulates the mass concentration of PM_2.5_, but without considering the source, size distribution, or chemical composition of those particles. The results presented in this study have several policy implications associated with RWC. These include a need for stricter guidelines for air quality, emission reduction measures, and improvement of pre-processing, storage, and combustion practices in order to decrease the associated health impacts. The implemented health impact assessment (HIA) methodology has been and will be a valuable tool of assessment for quantifying the health effects associated with exposure to RWC.

## Data Availability

The data that support the findings of this study are available from the corresponding author, [H.O.], upon reasonable request.

## Abbreviations

CI: confidence interval
CO: carbon monoxide
CRF: concentration-response function
HIA: health impact assessment
HR: hazard ratio
OR: odds ratio
PAH: polycyclic aromatic hydrocarbons
PM_2.5_: fine particles
PM_10_: particulate matter
RR: risk ratio
RWC: residential wood combustion
SD: standard deviation
SSYK: Swedish Standard Classification of Occupation
VOC: volatile organic hydrocarbons
WHO: World Health Organization
YLL: years of life lost

## References

[1] Lewis C (1981). Biomass through the ages. Biomass.

[2] Vakkilainen E, Kuparinen K, Heinimö J. Large Industrial Users of Energy Biomass. In. Lappeenranta Lappeenranta University of Technology; 2013.

[3] Vicente ED, Alves CA (2018). An overview of particulate emissions from residential biomass combustion. Atmos Res.

[4] World Health Organization European Centre for Environment. WHO global air quality guidelines: particulate matter (PM2.5 and PM10), ozone, nitrogen dioxide, sulfur dioxide and carbon monoxide. Geneva: World Health Organization; 2021. 34662007

[5] Landrigan PJ, Fuller R, Acosta NJR, Adeyi O, Arnold R, Basu NN, Baldé AB, Bertollini R, Bose-O’Reilly S, Boufford JI, et al. The Lancet Commission on pollution and health. Lancet (London, England) 2018, 391(10119):462–512. 10.1016/S0140-6736(17)32345-0 29056410

[6] et al: Global burden of 87 risk factors in 204 countries and territories, 1990–2019:a systematic analysis for the Global Burden of Disease Study 2019. Lancet. 2020; 396(10258):1223–1249. 10.1016/S0140-6736(20)30752-2 PMC7566194 33069327

[7] Beelen R, Raaschou-Nielsen O, Stafoggia M, Andersen ZJ, Weinmayr G, Hoffmann B, Wolf K, Samoli E, Fischer P, Nieuwenhuijsen M (2014). Effects of long-term exposure to air pollution on natural-cause mortality: an analysis of 22 European cohorts within the multicentre ESCAPE project. The Lancet.

[8] Orellano P, Reynoso J, Quaranta N, Bardach A, Ciapponi A (2020). Short-term exposure to particulate matter (PM(10) and PM(2.5)), nitrogen dioxide (NO(2)), and ozone (O(3)) and all-cause and cause-specific mortality: Systematic review and meta-analysis. Environ Int.

[9] Chen J, Li C, Ristovski Z, Milic A, Gu Y, Islam MS, Wang S, Hao J, Zhang H, He C (2017). A review of biomass burning: Emissions and impacts on air quality, health and climate in China. Sci Total Environ.

[10] Olsen Y, Nøjgaard JK, Olesen HR, Brandt J, Sigsgaard T, Pryor SC, Ancelet T, Viana MdM, Querol X, Hertel O (2020). Emissions and source allocation of carbonaceous air pollutants from wood stoves in developed countries: A review. Atmospheric Pollution Research.

[11] Cincinelli A, Guerranti C, Martellini T, Scodellini R (2019). Residential wood combustion and its impact on urban air quality in Europe. Curr Opin Environ Sci Health.

[12] Flach B, Lieberz S, Bolla S. Biofuels Annual. Report Number: E42020-0032. In. The Hague: Global Agricultural Information Network 2020.

[13] Naeher LP, Brauer M, Lipsett M, Zelikoff JT, Simpson CD, Koenig JQ, Smith KR (2007). Woodsmoke health effects: a review. Inhalation Toxicol.

[14] Boman BC, Forsberg AB, Järvholm BG (2003). Adverse health effects from ambient air pollution in relation to residential wood combustion in modern society. Scand J Work Environ Health.

[15] Kleeman MJ, Schauer JJ, Cass GR (1999). Size and Composition Distribution of Fine Particulate Matter Emitted from Wood Burning, Meat Charbroiling, and Cigarettes. Environ Sci Technol.

[16] Trojanowski R, Fthenakis V (2019). Nanoparticle emissions from residential wood combustion: A critical literature review, characterization, and recommendations. Renew Sustain Energy Rev.

[17] Butt EW, Rap A, Schmidt A, Scott CE, Pringle KJ, Reddington CL, Richards NAD, Woodhouse MT, Ramirez-Villegas J, Yang H (2016). The impact of residential combustion emissions on atmospheric aerosol, human health, and climate. Atmos Chem Phys.

[18] Karagulian F, Belis CA, Dora CFC, Prüss-Ustün AM, Bonjour S, Adair-Rohani H, Amann M (2015). Contributions to cities’ ambient particulate matter (PM): A systematic review of local source contributions at global level. Atmos Environ.

[19] Johnston HJ, Mueller W, Steinle S, Vardoulakis S, Tantrakarnapa K, Loh M, Cherrie JW (2019). How Harmful Is Particulate Matter Emitted from Biomass Burning? A Thailand Perspective. Curr Pollution Rep.

[20] Oudin A, Segersson D, Adolfsson R, Forsberg B (2018). Association between air pollution from residential wood burning and dementia incidence in a longitudinal study in Northern Sweden. PLoS ONE.

[21] Barregard L, Sällsten G, Gustafson P, Andersson L, Johansson L, Basu S, Stigendal L (2006). Experimental exposure to wood-smoke particles in healthy humans: effects on markers of inflammation, coagulation, and lipid peroxidation. Inhalation Toxicol.

[22] Walker ES, Fedak KM, Good N, Balmes J, Brook RD, Clark ML, Cole-Hunter T, Devlin RB, L’Orange C, Luckasen G (2022). Acute differences in blood lipids and inflammatory biomarkers following controlled exposures to cookstove air pollution in the STOVES study. Int J Environ Health Res.

[23] Fedak KM, Good N, Walker ES, Balmes J, Brook RD, Clark ML, Cole-Hunter T, Devlin R, L’Orange C, Luckasen G (2020). Acute changes in lung function following controlled exposure to cookstove air pollution in the subclinical tests of volunteers exposed to smoke (STOVES) study. Inhalation Toxicol.

[24] Cole-Hunter T, Dhingra R, Fedak KM, Good N, L’Orange C, Luckasen G, Mehaffy J, Walker E, Wilson A, Balmes J (2021). Short-term differences in cardiac function following controlled exposure to cookstove air pollution: The subclinical tests on volunteers exposed to smoke (STOVES) study. Environ Int.

[25] Schwartz C, Bølling AK, Carlsten C (2020). Controlled human exposures to wood smoke: a synthesis of the evidence. Part Fibre Toxicol.

[26] Sigsgaard T, Forsberg B, Annesi-Maesano I, Blomberg A, Bølling A, Boman C, Bønløkke J, Brauer M, Bruce N, Héroux ME (2015). Health impacts of anthropogenic biomass burning in the developed world. Eur Respir J.

[27] Grahame TJ, Klemm R, Schlesinger RB (2014). Public health and components of particulate matter: The changing assessment of black carbon. J Air Waste Manag Assoc.

[28] Janssen NA, Hoek G, Simic-Lawson M, Fischer P, van Bree L, ten Brink H, Keuken M, Atkinson RW, Anderson HR, Brunekreef B, et al. Black carbon as an additional indicator of the adverse health effects of airborne particles compared with PM10 and PM2.5. Environ Health Perspect. 2011;119(12):1691–1699. 10.1289/ehp.1003369 PMC3261976 21810552

[29] Vodonos A, Awad YA, Schwartz J (2018). The concentration-response between long-term PM(2.5) exposure and mortality; A meta-regression approach. Environ Res.

[30] Johnston FH, Hanigan IC, Henderson SB, Morgan GG. Evaluation of interventions to reduce air pollution from biomass smoke on mortality in Launceston, Australia: retrospective analysis of daily mortality, 1994–2007. BMJ. 2013;346:e8446. 10.1136/bmj.e8446 PMC3541469 23299843

[31] Kukkonen J, López-Aparicio S, Segersson D, Geels C, Kangas L, Kauhaniemi M, Maragkidou A, Jensen A, Assmuth T, Karppinen A (2020). The influence of residential wood combustion on the concentrations of PM2.5 in four Nordic cities. Atmos Chem Phys.

[32] Plejdrup MS, Nielsen O-K, Brandt J (2016). Spatial emission modelling for residential wood combustion in Denmark. Atmos Environ.

[33] HSY. Population grid of Helsinki Metropolitan Area. Helsinki Region Environmental Services. In.: Helsinki Region Infoshare service; 2020.

[34] SCB. Deaths by region, age (the year of birth) and sex. In.: Statistics Sweden; 2020.

[35] Tilastokeskus. Deaths by age (5-year), sex and area. In.: Statistics Finland; 2020.

[36] Statistisk sentralbyra. Deaths, by sex and 10-year age groups. In.: Statistics Norway; 2020.

[37] Statistikbanken: Deaths by region, sex, age and cause of death. In.: Statistics Denmark; 2020.

[38] Hvidtfeldt UA, Sørensen M, Geels C, Ketzel M, Khan J, Tjønneland A, Overvad K, Brandt J, Raaschou-Nielsen O (2019). Long-term residential exposure to PM(2.5), PM(10), black carbon, NO(2), and ozone and mortality in a Danish cohort. Environ Int.

[39] Turner MC, Jerrett M, Pope CA, Krewski D, Gapstur SM, Diver WR, Beckerman BS, Marshall JD, Su J, Crouse DL (2016). Long-Term Ozone Exposure and Mortality in a Large Prospective Study. Am J Respir Crit Care Med.

[40] WHO. AirQ+: software tool for health risk assessment of air pollution. In.; 2021.

[41] Lehtomäki H, Geels C, Brandt J, Rao S, Yaramenka K, Åström S, Andersen MS, Frohn LM, Im U, Hänninen O (2020). Deaths attributable to air pollution in Nordic Countries: Disparities in the estimates. Atmosphere.

[42] WHO. Review of evidence on health aspects of air pollution - REVIHAAP Project: Technical Report. In. Copenhagen: WHO Regional Office for Europe; 2013. 27195369

[43] Vohra K, Vodonos A, Schwartz J, Marais EA, Sulprizio MP, Mickley LJ (2021). Global mortality from outdoor fine particle pollution generated by fossil fuel combustion: Results from GEOS-Chem. Environ Res.

[44] Savolahti M, Lehtomäki H, Karvosenoja N, Paunu VV, Korhonen A, Kukkonen J, Kupiainen K, Kangas L, Karppinen A, Hänninen O. Residential Wood Combustion in Finland: PM(2.5) Emissions and Health Impacts with and without Abatement Measures. Int J Environ Res Public Health. 2019;16(16):2920. 10.3390/ijerph16162920 PMC6719946 31416284

[45] Kukkonen J, Kangas L, Kauhaniemi M, Sofiev M, Aarnio M, Jaakkola JJK, Kousa A, Karppinen A (2018). Modelling of the urban concentrations of PM2.5 on a high resolution for a period of 35 years, for the assessment of lifetime exposure and health effects. Atmos Chem Phys.

[46] Stafoggia M, Oftedal B, Chen J, Rodopoulou S, Renzi M, Atkinson RW, Bauwelinck M, Klompmaker JO, Mehta A, Vienneau D (2022). Long-term exposure to low ambient air pollution concentrations and mortality among 28 million people: results from seven large European cohorts within the ELAPSE project. Lancet Planet Health.

[47] Chen J, Hoek G (2020). Long-term exposure to PM and all-cause and cause-specific mortality: A systematic review and meta-analysis. Environ Int.

[48] Gustafsson M, Lindén J, Tang L, Forsberg B, Orru H, Åström S, Sjöberg K. Quantification of population exposure to NO2, PM2. 5 and PM10 and estimated health impacts. In.; 2018.

[49] Jerrett M, Burnett RT, Ma R, Pope CA, Krewski D, Newbold KB, Thurston G, Shi Y, Finkelstein N, Calle EE (2005). Spatial analysis of air pollution and mortality in Los Angeles. Epidemiol (Cambridge Mass).

[50] Díaz-Robles L, Cortés S, Vergara-Fernández A, Ortega JC (2015). Short Term Health Effects of Particulate Matter: A Comparison between Wood Smoke and Multi-Source Polluted Urban Areas in Chile. Aerosol Air Qual Res.

[51] Pindus M, Orru H, Maasikmets M, Kaasik M, Jõgi R (2016). Association Between Health Symptoms and Particulate Matter from Traffic and Residential Heating - Results from RHINE III in Tartu. The open respiratory medicine journal.

[52] Riddervold IS, Bønløkke JH, Olin AC, Grønborg TK, Schlünssen V, Skogstrand K, Hougaard D, Massling A, Sigsgaard T (2012). Effects of wood smoke particles from wood-burning stoves on the respiratory health of atopic humans. Part Fibre Toxicol.

[53] Forchhammer L, Møller P, Riddervold IS, Bønløkke J, Massling A, Sigsgaard T, Loft S (2012). Controlled human wood smoke exposure: oxidative stress, inflammation and microvascular function. Part Fibre Toxicol.

[54] Karvosenoja N, Kangas L, Kupiainen K, Kukkonen J, Karppinen A, Sofiev M, Tainio M, Paunu V-V, Ahtoniemi P, Tuomisto JT, et al. Integrated modeling assessments of the population exposure in Finland to primary PM2.5 from traffic and domestic wood combustion on the resolutions of 1 and 10 km. Air Quality Atmosphere Health. 2011; 4(3):179–188.

[55] Korhonen A, Lehtomäki H, Rumrich I, Karvosenoja N, Paunu V-V, Kupiainen K, Sofiev M, Palamarchuk Y, Kukkonen J, Kangas L (2019). Influence of spatial resolution on population PM2.5 exposure and health impacts. Air Qual Atmos Health.

[56] Swab C, Allen P, Armitage S, Biberic A (2019). 2014 residential wood combustion survey: Results overview and spatial allocation of emissions estimates. Atmos Environ.

[57] Chakraborty R, Heydon J, Mayfield M, Mihaylova L (2020). Indoor Air Pollution from Residential Stoves: Examining the Flooding of Particulate Matter into Homes during Real-World Use. Atmosphere.

[58] Piccardo MT, Cipolla M, Stella A, Ceppi M, Bruzzone M, Izzotti A, Valerio F (2014). Indoor pollution and burning practices in wood stove management. J Air Waste Manag Assoc.

[59] Vicente ED, Vicente AM, Evtyugina M, Oduber FI, Amato F, Querol X, Alves C (2020). Impact of wood combustion on indoor air quality. Sci Total Environ.

[60] Groot J, Keller A, Pedersen M, Sigsgaard T, Loft S, Nybo Andersen A-M (2022). Indoor home environments of Danish children and the socioeconomic position and health of their parents: A descriptive study. Environ Int.

[61] Laursen KR, Rasmussen BB, Rosati B, Gutzke VH, Østergaard K, Ravn P, Kjærgaard SK, Bilde M, Glasius M, Sigsgaard T (2021). Acute health effects from exposure to indoor ultrafine particles—A randomized controlled crossover study among young mild asthmatics. Indoor Air.

[62] Price-Allison A, Lea-Langton AR, Mitchell EJS, Gudka B, Jones JM, Mason PE, Williams A (2019). Emissions performance of high moisture wood fuels burned in a residential stove. Fuel.

[63] Soares J, Kousa A, Kukkonen J, Matilainen L, Kangas L, Kauhaniemi M, Riikonen K, Jalkanen JP, Rasila T, Hänninen O (2014). Refinement of a model for evaluating the population exposure in an urban area. Geosci Model Dev.

[64] Shen G, Xue M, Wei S, Chen Y, Zhao Q, Li B, Wu H, Tao S (2013). Influence of fuel moisture, charge size, feeding rate and air ventilation conditions on the emissions of PM, OC, EC, parent PAHs, and their derivatives from residential wood combustion. J Environ Sci.

[65] Fachinger F, Drewnick F, Gieré R, Borrmann S (2017). How the user can influence particulate emissions from residential wood and pellet stoves: Emission factors for different fuels and burning conditions. Atmos Environ.

[66] Tytgat T, Walpot G, Cools J, Lenaerts S. Literature review of emissions of modern wood combustion devices and emissions reducing technologies, under real-life conditions. University of Antwerp, Rapport voor Vlaamse Milieu Maatschappij 2017.

[67] Orru K, Nordin S, Harzia H, Orru H: The role of perceived air pollution and health risk perception in health symptoms and disease: a population-based study combined with modelled levels of PM(10). Int Arch Occupational Environ Health 2018;91(5):581–589. 10.1007/s00420-018-1303-x PMC6002462 29602966

[68] Schlesinger WH (2018). Are wood pellets a green fuel?. Sci (New York NY).

[69] Corsini E, Marinovich M, Vecchi R (2019). Ultrafine Particles from Residential Biomass Combustion: A Review on Experimental Data and Toxicological Response. Int J Mol Sci.

[70] Meyer NK (2012). Particulate, black carbon and organic emissions from small-scale residential wood combustion appliances in Switzerland. Biomass Bioenergy.

[71] Arvesen A, Cherubini F, del Alamo Serrano G, Astrup R, Becidan M, Belbo H, Goile F, Grytli T, Guest G, Lausselet C (2018). Cooling aerosols and changes in albedo counteract warming from CO2 and black carbon from forest bioenergy in Norway. Sci Rep.

[72] Hellén H, Kangas L, Kousa A, Vestenius M, Teinilä K, Karppinen A, Kukkonen J, Niemi JV (2017). Evaluation of the impact of wood combustion on benzo[a]pyrene (BaP) concentrations; ambient measurements and dispersion modeling in Helsinki, Finland. Atmos Chem Phys.

[73] EEA. Air quality in Europe — 2020 report. In. Luxembourg: European Environment Agency; 2020.

[74] Wheeler AJ, Gibson M, Ward T, Allen RW, Guernsey JR, Seaboyer M, Kuchta J, Gould R, Stieb D: Reductions in residential wood smoke concentrations and infiltration efficiency using electrostatic air cleaner interventions. In: 12th International Conference on Indoor Air Quality and Climate: 2011; 2011: 5–10.

[75] Levander T, Bodin S (2014). Controlling Emissions from Wood Burning: Legislation and Regulations in Nordic Countries to Control Emissions from Residential Wood Burning An examination of Past Experience.

[76] Allen RW, Leckie S, Millar G, Brauer M (2009). The impact of wood stove technology upgrades on indoor residential air quality. Atmos Environ.

[77] Jeong C-H, Evans GJ, Dann T, Graham M, Herod D, Dabek-Zlotorzynska E, Mathieu D, Ding L, Wang D (2008). Influence of biomass burning on wintertime fine particulate matter: Source contribution at a valley site in rural British Columbia. Atmos Environ.

[78] Lopez-Aparicio S, Grythe H (2020). Evaluating the effectiveness of a stove exchange programme on PM2.5 emission reduction. Atmos Environ.

[79] Noonan CW, Ward TJ, Navidi W, Sheppard L (2012). A rural community intervention targeting biomass combustion sources: effects on air quality and reporting of children’s respiratory outcomes. Occup Environ Med.

